# Effective Method for a Graphene Oxide with Impressive Selectivity in Carboxyl Groups

**DOI:** 10.3390/nano12183112

**Published:** 2022-09-08

**Authors:** Iluminada Rodríguez-Pastor, Adelia López-Pérez, María D. Romero-Sánchez, Juana M. Pérez, Ignacio Fernández, Ignacio Martin-Gullon

**Affiliations:** 1 Applynano Solutions S.L., Alicante Scientific Park #3, 03690 Alicante, Spain; 2 Institute of Chemical Processes Engineering, University of Alicante, 03080 Alicante, Spain; 3 Research Centre CIAIMBITAL, University of Almería, 04120 Almería, Spain

**Keywords:** reduced graphene oxide, graphite intercalated compound, carboxyl groups, bioconjugation

## Abstract

The development of new applications of graphene oxide in the biomedical field requires the covalent bonding of bioactive molecules to a sheet skeleton. Obtaining a large carboxyl group population over the surface is one of the main targets, as carboxyl group concentration in conventional graphene oxide is low among a majority of non-useful sp3-C-based functionalities. In the present work, we propose a selective method that yields an impressive increase in carboxyl group population using single-layer, thermally reduced graphene oxide as a precursor in a conventional Hummers–Offemann reaction. When starting with a reduced graphene oxide with no interlayer registry, sulfuric acid cannot form a graphite intercalated compound. Then, potassium permanganate attacks in in-plane (vacancies or holes) structural defects, which are numerous over a thermally reduced graphene oxide, as well as in edges, yielding majorly carboxyl groups without sheet cutting and unzipping, as no carbon dot formation was observed. A single-layer precursor with no ordered stacking prevents the formation of an intercalated compound, and it is this mechanism of the potassium permanganate that results in carboxyl group formation and the hydrophilic character of the compound.

## 1. Introduction

Graphene oxide (GO), a single and separated layer derived from graphite oxide (GrO), is a form of graphene based-material with considerable industrial applicability, especially adequate for applications that require large-scale amounts, such us energy storage [1], multifunctional polymer nanocomposites [2] and, more recently, applications related to the biomedical field, such as antimicrobial [3], biosensing [4], bioimaging [5] and drug-delivery applications [6]. In particular, it of key importance in drug delivery (for use in vivo) to obtain an optimized dispersion and compatibility of GO with the media (polymeric, water or solvent-based media) in which it is incorporated. Therefore, specific and selective chemical functionalization of the GO surface is required [7]. It is generally accepted that GO presents a non-stoichiometric structure based on the Lerf–Klinowski model [8], with aromatic isolated domains in the basal plane in a continuous chain of sp3 carbons with epoxide and hydroxyl groups, as well as carboxyls, carboxylic anhydrides, quinones, lactones and lactols [9] on the edges and internal defects (such as holes) of the graphene sheet. However, the structure is very complex and remains to be fully elucidated [10], and there is an intense debate about the mechanism of graphite/graphene oxide formation through the Hummers–Offemann method [11] (potassium permanganate reaction with an H_2_SO_4_-based graphite intercalated compound (GIC) [12]), the reaction conditions [13] and the resulting structure in terms of poorly oxidated GO layers and small entities of oxidative debris or fulvic substances [14,15] as demonstrated by the oxidation of multiwall carbon nanotubes [16,17]. For the development of most of the biomedical applications, bioactive molecules must be covalently linked through GO oxygenated groups (i.e., bioconjugation), with carboxyl groups being the most convenient target, although their population over GO surfaces is low [18]. Therefore, a more selective formation of carboxyl groups and a reduction in the amount of other oxygen moieties is desirable in order to obtain a GO with useful carboxylic oxygen to conjugate with biomolecules.

The main challenge is to efficiently increase the amount of carboxyl groups by either the conversion of hydroxyls or epoxies or by generating new carboxylic groups while removing existing sp3C-O groups. Jiříčková et al. [19] reported a double-step oxidation process based on the Tour method (KMnO_4_ in H_2_SO_4_-H_3_PO_4_) [20], yielding a material with a layer stacking typical of a graphite oxide and a broad peak in the XPS C1s spectrum centered at 288 eV, ranging from 283 to 294 eV, indicative of extensive oxidation, with a major population of C-O bonds relative to that of C-C bonds and, semiquantitatively, a higher contribution of carbonylic/carboxylic groups with respect to sp3-C-O bonds and no specific selectivity for carboxylic groups. Another reported method is GO treatment with chloroacetic acid in high alkaline NaOH water-based conditions as an intermediate and successful step for conjugation with bioactive molecules [21]. Imani et al. [22] reported an optimal concentration of chloroacetic acid as high as 2M, attaining zeta potentials as low as −45 mV and a nearly complete loss of the 002 interlayer registry by XRD. Single NaOH treatment on GO results in appreciable oxygen removal, which can be explained by either hydroxyl reduction in the presence of an epoxy ring [23] or simple separation of fulvic substances [14,24], yielding GO surface sheets with a small but clear contribution of carboxyl groups identified by XPS [10,14]. More recently, Guo et al. [25] indicated that high NaOH concentrations of 3M are required for an appreciable carboxylation effect of chloroacetic acid, highlighting a combined mechanism of hydroxyl group reduction and epoxide ring opening evolution to carboxyl groups, leading a final GO with an enhanced and clear contribution of carboxylic moieties (17% of C-O functionalities determined by a 288.6 eV deconvolution peak in the C1s XPS plot) but still a majority (81%) of sp3C-O bonds. With respect to other carboxylation methods, Kuang et al. [26] reported a treatment with 1-pyrenecarboxaldehyde in diluted NaOH, with an appreciable reduction in sp3C-O content while maintaining sp2C=O groups and increasing carboxylic groups to 20.4% of total C-O bonds. Pan et al. [27] increased the population of carboxylic acids by thiourea dioxide treatment in aqueous ammonia, enhancing the relative carboxyl population to 30% with respect to other oxygen moieties, considerably reducing decomposition weight loss and attaining a loss of registry in the 002 XRD peak indicative of a single-layer carboxyl GO.

As previously stated, the oxidation mechanism of graphitic materials by potassium permanganate in sulfuric acid is still not completely clear. Most evidence was obtained by starting with multiwall carbon nanotubes (MWCNTs), whereby graphene shells are unequivocally unraveled due to an unzipping of the graphene layer [28]. The graphene unzipping mechanism was reported to be oxygen-driven through the opening of epoxy rings placed in an adequate arrangement [29], although DFT studies indicated that this oxygen-driven unzipping is limited by oxygen diffusion and mobility of the epoxy groups [30]. It has been reported that MWCNTs must have adequate diameters (inner space of more than 20 nm) for an effective unravelling of all the shells [31,32]; otherwise, the unravelling of all the shells is not complete. Accordingly, Dimiev et al. [33] pointed out that the unzipping of graphene sheets in MWCNTs by KMnO_4_ and H_2_SO_4_ must be conducted by an intercalation-driven mechanism, valid in wider shells, where sulfuric acid adequately forms an intercalated compound. General knowledge in organic chemistry indicates that potassium permanganate does not react with isolated aromatic rings [34] but might form carbonyls in activated alkyl groups close to aromatic rings and oxidize localized double C=C bonds, producing diols in a former step that may evolve into cleavage-generating carboxylic acids on each carbon.

The objective of the present work is to selectively form carboxyl groups over the surface of a single-layer reduced graphene oxide (rGO) by potassium permanganate in sulfuric acid. rGO is an exfoliated material; therefore, it contains no stacked graphene-derived layers, and consequently, no intercalation is produced with H_2_SO_4_. As the Hummers–Offemann reaction mechanism for unzipping graphene layers is claimed to be intercalation-driven, the hypothesis of the present work involved the use of an exfoliated material as precursor, such as rGO, which cannot form an intercalated compound (GIC), and to produce different forms of KMnO_4_ oxidation over a graphene-based sheet with plenty of defects and localized double bonds without unzipping of the layer. Over this heterogeneous structure of thermally reduced graphene oxide, a considerable and selective carboxyl group population is formed.

## 2. Materials and Methods

### 2.1. Materials

Natural expanded graphite BNB90 was supplied by Imerys (Bodio, Switzerland). KMnO_4_, NaNO_3_, H_2_SO_4_ (95%) and HCl (37%) were supplied by VWR. H_2_O_2_ (33 vol%) was purchased from Fisher.

### 2.2. Preparation of Carboxyl GO (GO-COOH)

First, graphite oxide (GrO) was produced following a modified Hummers–Offemann method. Initially, 5 g of graphite, 350 mL of H_2_SO_4_ and 5 g of NaNO_3_ were mixed and stirred at room temperature. After 3 h, 20 g of KMnO_4_ was slowly added, and the suspension was stirred for 2 h. Subsequently, the temperature was increased to 55 °C and maintained for 1 h. Once the reaction was complete, the mixture was cooled to room temperature and poured into a 5L flask containing ice-cold water, inducing water solvation of the KMnO_4_/carbon reaction and yielding GrO. After 1 min, 40 mL of H_2_O_2_ (33 vol%) was added to stop KMnO_4_ oxidation, converting all manganese moieties to soluble Mn^2+^, quenching the KMnO_4_ oxidation and preventing MnO_2_ precipitation, leaving GrO as the only insoluble material. After filtration, the solid cake was washed with 50 mL of HCl (20 vol%) for 30 min with stirring and repeated filtering. Finally, washing and filtration were repeated with H_2_O (100 mL). A drying treatment in a vacuum oven at 70 °C overnight yielded GrO. The GrO yield was 200.1 wt% with respect to the starting graphite. This value or more than 100% is a clear indication of excellent oxygen uptake during GrO formation and interlayer chemisorbed water, as previously reported [27]. Drying treatment is regularly carried out below 100 °C under mild vacuum conditions to prevent thermal expansion.

GrO was thermally reduced and exfoliated through a microwave treatment [35], yielding reduced graphene oxide (rGO). Then, 1 g of GrO was placed in a PTFE digestor and directly vented in a Milstone ULTRAwave microwave furnace for 90 s at 800 W of power. Most of the fluffy rGO powder was collected using a homemade glass-fiber filtering medium. The rGO partial yield was 40.5 wt% with respect to the starting GrO (81.1 wt% with respect to graphite).

Finally, the rGO sample was selectively carboxylated with an oxidation treatment based on the Hummers–Offemann method. Initially, 1 g of rGO was poured into 200 mL of H_2_SO_4_ with 1 g of NaNO_3_ and stirred for 3 h. Subsequently, 4 g of KMnO_4_ was slowly added to the suspension and stirred for 2 h. The temperature was increased to 55 °C and maintained for 1 hour. As previously described, the mixture was cooled down to room temperature and poured into a 500-mL flask with ice water, adding 40 mL H_2_O_2_ (20 vol%) one minute after. Finally, the reaction mixture was filtered, washed with 50 mL HCl (20%) for 30 min and filtered again. Washing and filtration were repeated with demineralized water, and the solid cake was dried in a vacuum oven at 70ºC overnight, yielding GO-COOH. The GO-COOH yield was 58.5 % with respect to rGO (47.4 wt% with respect to graphite), indicating a net weight loss in this step and, consequently, a carbon loss.

### 2.3. Characterization

Transmission electron microscopy (TEM) images were obtained on a JEOL (model JEM-1400 Plus equipped with an image acquisition camera, model GATAN); isopropanol was used as solvent to disperse the solid samples at 0.1 mg/mL using an ultrasonic tip (30 W, 1 h with ON-OFF intervals of 60–30 s). In the case of the GrO sample, it exfoliates to GO, whereas rGO and GO-COOH are already exfoliated prior to sonication. A drop of suspension was deposited on a carbon-coated copper grid, evaporating the solvent at room temperature. Zeta potential was determined using a Nanotrac Flex (Verder) combined with Stabino (Colloid Metrics) for water suspensions. Water suspensions were prepared by diluting 0.01 mg/mL from a parent suspension at 1 mg/mL, which was horn-sonicated (30 W, 2 h with ON-OFF intervals of 60–30 s). Suspensions of GO and GO-COOH were measured.

GrO, rGO and GO-COOH were characterized in powder form by X-ray diffraction (XRD), X-ray photoelectron spectroscopy (XPS), infrared spectroscopy with attenuated total reflectance (IR-ATR), ^13^C nuclear magnetic resonance (^13^C-NMR) spectroscopy, Raman spectroscopy and thermogravimetric analysis coupled with mass spectrometry (TGA-MS). XRD was carried out using a Bruker D8-Advance device, and acquisition was achieved with a step of 0.05°. XPS was carried out with a K-alpha spectrometer (Thermo-Scientific); the surface atomic O/C ratio was calculated by integrating the spectra, and analysis of functional groups bonded to C was performed by deconvoluting the C_1s_ spectra to 5 Voigt functions (70% Gaussian, 30% Lorentzian) centered at 284.6, 285.6, 286.5, 287.6 and 288.7 eV to quantify sp2C=sp2C, sp3C-sp3C, sp3C-O (hydroxyl, epoxy), sp2C=O and sp2C(O)OH, respectively, according the criteria of Pan et al. [27], which are compatible for comparison with other carboxylation methods in the literature [25,26]. Three XPS spectra in different zones of the sample were acquired with satisfactory reproducibility. IR-ATR was performed with a BRUKER IFS 66 infrared instrument, using an ATR attachment. Solid-state ^13^C magic-angle spinning NMR spectra (^13^C MAS NMR) were acquired on a Bruker Avance-600 spectrometer (150.9 MHz ^13^C, 600.1 MHz ^1^H) using the HPDEC Bruker pulse program with 10 kHz MAS, a spectral width of 90,909 Hz (600 ppm), a recycle delay of 12 s and a 1.5 µs pulse with 3000 scans. Raman spectroscopy was performed with a Thermo Scientific NEXSA instrument, using a 532 nm laser. Baseline was subtracted, and spectra were represented normalized to the G peak. TG-MS was performed using a Mettler Toledo apparatus (TGA/SDT851e/LF/1600 coupled with a Thermostat GSD301T) in order to measure the weight loss and gas evolution upon thermal decomposition of the samples; runs were carried out under a helium atmosphere, from room temperature to 1000 ºC at a rate of 10 °C/min, with *m/z* = 18 (H_2_O), 28 (CO) and 44 (CO_2_), which were integrated and quantified using a CaC_2_O_4_·H_2_O standard. In addition, *m/z* = 64 (SO_2_) was monitored but not quantified.

## 3. Results

**Figure 1:**
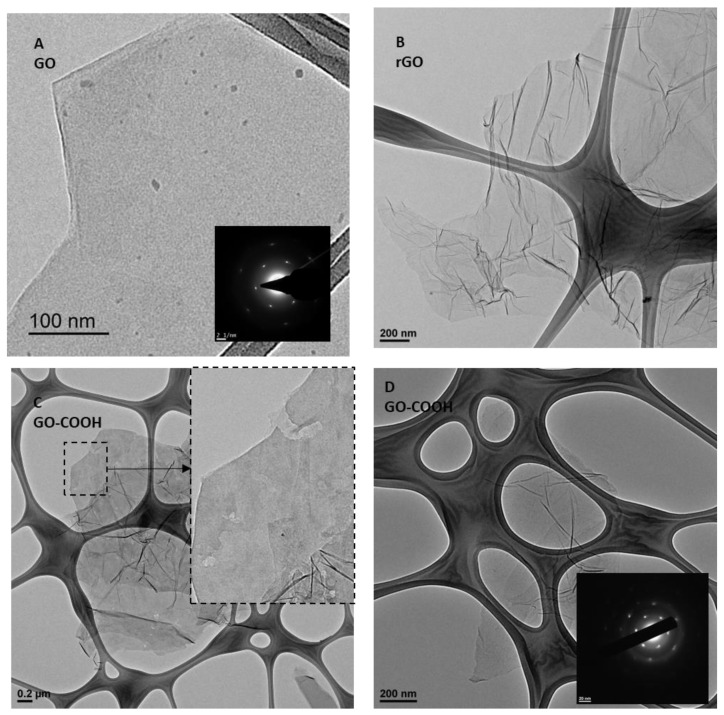
(**A**) Single-layer GO sheet from GrO exfoliated by liquid-phase sonication; the morphology is flat without visible damage. (**B**) Thermally microwave-exfoliated rGO, showing roughness. (**C**) Wrinkled GO-COOH sheet, presenting with very clear damage and holes in both edges and the basal plane. (**D**) Wrinkled GO-COOH sheet with a flat zone, which indicates a single-layer according to its EDP (insert).

Figure 1 shows a selection of TEM exploration pictures of the three samples. Figure 1A shows an image of a single sheet from a GrO sample exfoliated into GO, as samples were adequately horn-sonicated in isopropanol for placement on the grid. This is a representative sheet of sample that is flat with straight edges and no visible holes or damaged zones on the basal plane, besides typical dots throughout the sheet corresponding to oxidative debris or fulvic compounds [36]. The electron diffraction pattern (EDP) shown in the inset in Figure 1A corresponds to a single-layer GO. A representative sheet of microwave-treated rGO sample is shown in Figure 1B, with many wrinkled planes, as generally reported for thermally reduced graphene oxide, including microwave-treated rGO [35]. This representative sheet has no visible damage in the basal plane; however it is expected to have large amounts of defects generated during the removal of oxygen groups removal during thermally decomposition and exfoliation of GrO [37]. Figure 1C,D show GO-COOH samples with appreciable differences. GO-COOH sheets are still quite wavy and wrinkled, like the parent rGO. However, the morphology in the sheet is highly damaged, as demonstrates clear holes across the sheets. On the other hand, no visible carbon dots are observed over the planes, so this second oxidation over an already thermally reduced flake differs considerably relative to existing GrO, without appreciable generation of fulvic compounds. In addition, some sheets present with flat zones, where an EDP could be achieved, as shown in Figure 1D; the inset shows a single-layer sheet. Additional photos are provided in the Supplementary Material (Figure S1).

**Figure 2:**
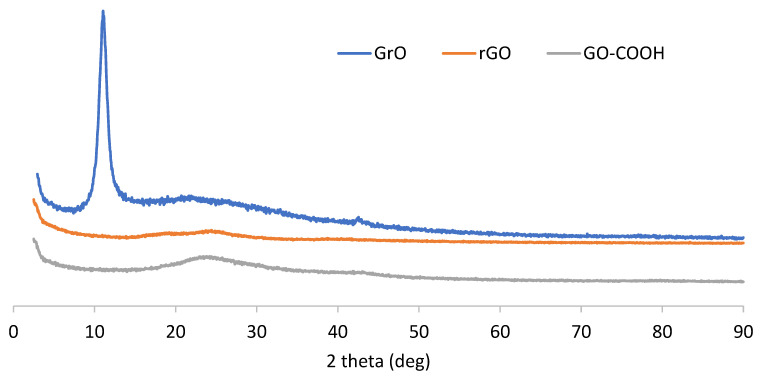
XRD diffractogram corresponding to GrO, rGO and GO-COOH powder samples.

Figure 2 shows the XRD diffractograms for the three synthetized samples; the Y axis reflects the absolute counts obtained for each diffraction run (only the baseline is moved for clarity of the plot). GrO presents with an XRD pattern typical of a graphite oxide, with a prominent peak at a 11.2° diffraction angle corresponding to the interlayer distance. Microwave-treated rGO yields a full disappearance of this peak, as expected, and there is no appreciable rearrangement of the layers associated with the graphite 002 peak at 26° (only a slight wave on the baseline, which is also the case for GrO), indicating a loss of interlayer registry. This is confirmed by the XRD pattern of the carboxyl GO-COOH sample, as no diffraction peak is observed at around 10° because the parent material (rGO) does not present graphitic stacked layers at around 26°. As a consequence, an oxidation treatment equivalent to a Hummers–Offemann reaction on a single-layer reduced graphene oxide must be mechanistically different from starting with a stacked graphitic material, as according to Dimiev and Tour [12], a first step of H_2_SO_4_-GIC formation occurs with further preliminary graphite oxide when adding KMnO_4_. With rGO, there is no GIC to form, as there is no crystalline sheet stacking. Some specific treatments of graphene oxide to produce carboxyl GO also yielded a loss of the interlayer registry, in particular, with chloroacetic acid/NaOH and thiourea dioxide [22,26].

**Figure 3:**
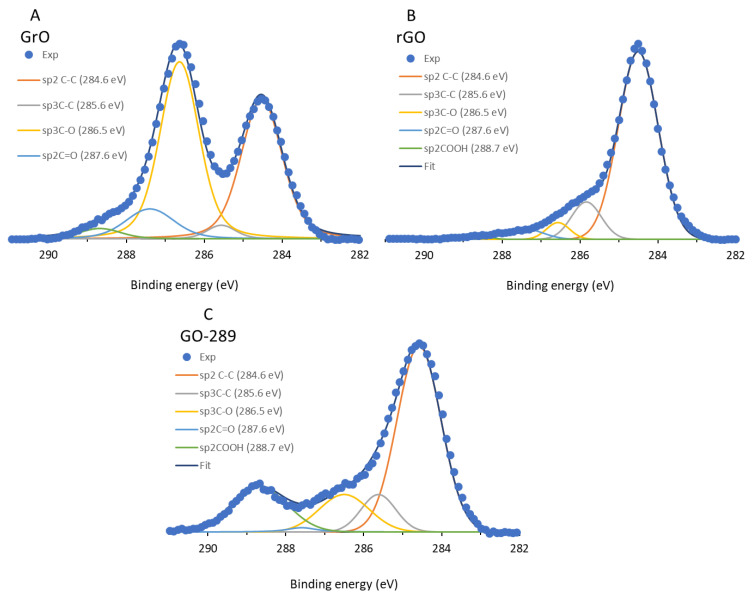
C1s XPS fitting plots for (**A**) GrO, (**B**) rGO and (**C**) GO-COOH.

GrO, rGO and GO-COOH powder samples were also characterized by XPS. Table 1 shows the surface elemental composition relative to the studied elements. On one hand, the survey spectrum does not indicate appreciable amounts of other elements. The C/O ratios for GrO and rGO are in accordance with those previously reported in the literature. On the other hand, carboxyl GO-COOH, with a treatment similar to the first oxidation of natural graphite, presents a higher C/O ratio. Figure 3 shows the C1s spectra for the three samples; the experimental counts were fitted to 5 main Voigt deconvolutions (70% Gaussian, 30% Lorentzian) centered at 284.6, 285.6, 286.5, 287.6 and 288.7 eV to quantify sp2C=sp2C, sp3C-sp3C, sp3C-O (hydroxyl, epoxy), sp2C=O and sp2C(O)OH, respectively, following the criteria of Pan et al. [27], which is similar to other methods and thus allows for quantitative comparison of the C1s contributions with other results reported in the literature.

Figure 3A shows the XPS C1s spectrum for GrO, which is typical for a graphite oxide; the main peak is located at 286.7 eV. Interpretations of which functional groups are included in this main and broad signal are numerous, but in this case, the signal might be ascribed to a main deconvolution centered at 286.5 eV, attributed to sp3C-O bonds, such as (from lower to higher energy) hydroxyl and ether-epoxy, and a minor deconvolution centered at 287.5 eV, attributed to lactols and sp2C quinone groups [10]. The next highest energy deconvolution peak was found at 288.7 eV, with low intensity, which is regularly attributed exclusively to carboxyl groups [38]. These carboxyls and carbonyls are present in a much lower extension than sp3C-O groups. Figure 3B shows the main contribution of the functional groups upon microwave treatment and conversion into rGO, (after the 284.6 eV deconvolution) at 285.6 eV, attributed to sp3C-sp3C bonds, and only small (or even negligible) contributions of ether/epoxy and carbonyls/carboxyls. A different situation occurs when oxidizing rGO with KMnO_4_ in sulfuric acid medium, as shown in Figure 3C. In this case, the main contribution is that at 284.6 eV of graphitic sp2-carbons, which was not the largest deconvolution contribution for GrO; the middle 286.5 eV deconvolution grows much less than that shown in Figure 3A, which is indicative of a clearly different oxidation mechanism. However, the most relevant issue is the significant contribution at 288.7 eV, which is characteristic of carboxyl groups with respect to other oxygen groups over the surface. To the best of our knowledge, this is the most selective XPS pattern to carboxyl groups ever reported for a graphene oxide [25,26,27], as quantified and shown in Table 2, with 18% of total C bonds and 55% of total C-O bonds. The carboxyl GO-COOH corresponds to micron-scale layers, with no dots or fulvic compounds. Table 2 shows a comparison of the present carboxylation method with others methods reported in the literature. In both chloroacetic acid and 1-pyrenecarboxaldehyde methods, the major contribution of C-O bonds corresponds to sp3C-O bonds (hydroxyl, ether and epoxy), whereas a carbonyl population dominates when using thiourea dioxide. However, carboxyl groups dominate the C-O surface when applying the Hummers–Offemann method to a single-layer rGO, with 55% of all oxygen moieties, representing a considerable increase with respect to the other reported methods. This major carboxyl population over the GO-COOH sample is also supported by the infrared spectra of the samples (see Figure S2) and ^13^C MAS NMR studies (see below), which confirm that carboxyl groups become much more numerous and hydroxyl groups decrease for GO-COOH with respect to parent GrO. Raman spectra (see Figure S3) present similar spectra for GrO and GO-COOH, which is indicative of a similar defect density.

**Figure 4:**
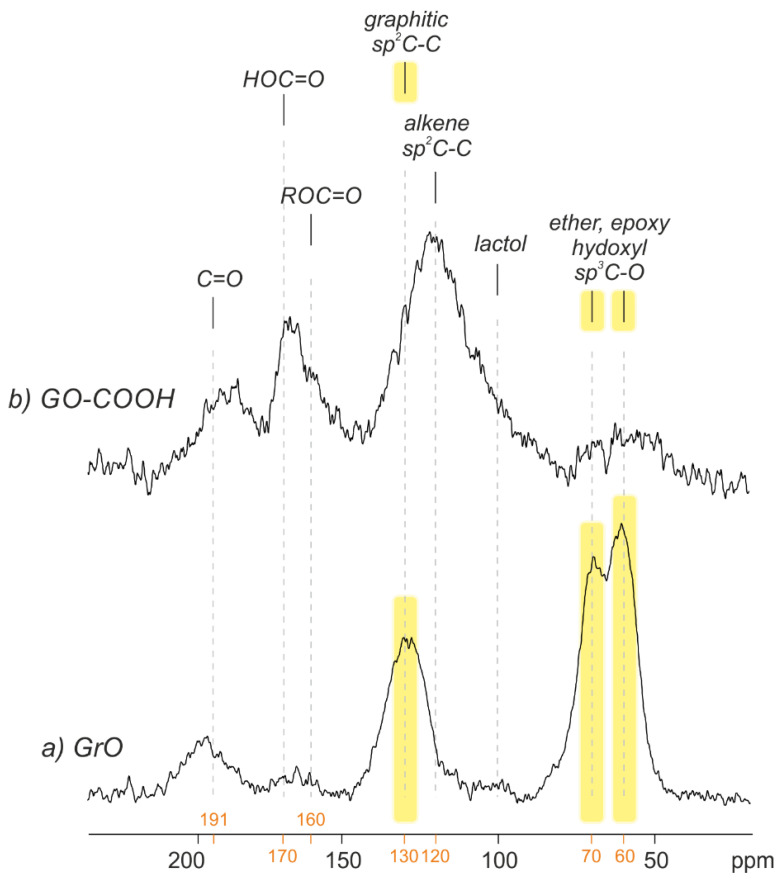
Direct-pulse ^13^C MAS NMR spectra of GrO and GO-COOH samples.

Figure 4 shows the ^13^C MAS NMR spectra corresponding to GrO and GO-COOH powdered samples (rGO yielded as low signal, as expected), which confirm XPS results. GrO presents a pattern typical of a graphite oxide, which is a key technique of the Lerf–Klinowski model [8], whereby the prominent peaks at 61 and 70 ppm are attributed to epoxy and hydroxyl groups, respectively; another prominent peak at 133 ppm is assigned to the remaining graphitic sp2 carbons; and a small signal at 191 ppm is assigned to carbonyls (ketone and aldehydes) [39,40]. The situation for GO-COOH is very different; nearly no 61 and 70 ppm peaks are detected, and a remarkable increase in carboxylic acids (170 ppm) is observed. In addition, there is an asymmetric peak centered at 120 ppm, which might correspond to a contribution of (i) graphitic sp2-C-C, which is normally located at 130 ppm (see Figure 4), (ii) localized C=C double bonds and (iii) lactol groups [9]. Undoubtedly, the reaction between KMnO_4_ in H_2_SO_4_ and single-layer rGO results in a completely different structure than when it is started with stacked graphite, with the lack of π-π stacking representing the main difference. The reaction mechanism cannot pass through an intercalation-driven process; therefore, the reactions are understood as organic chemistry reactions, which leave intact aromatic zones.

**Figure 5:**
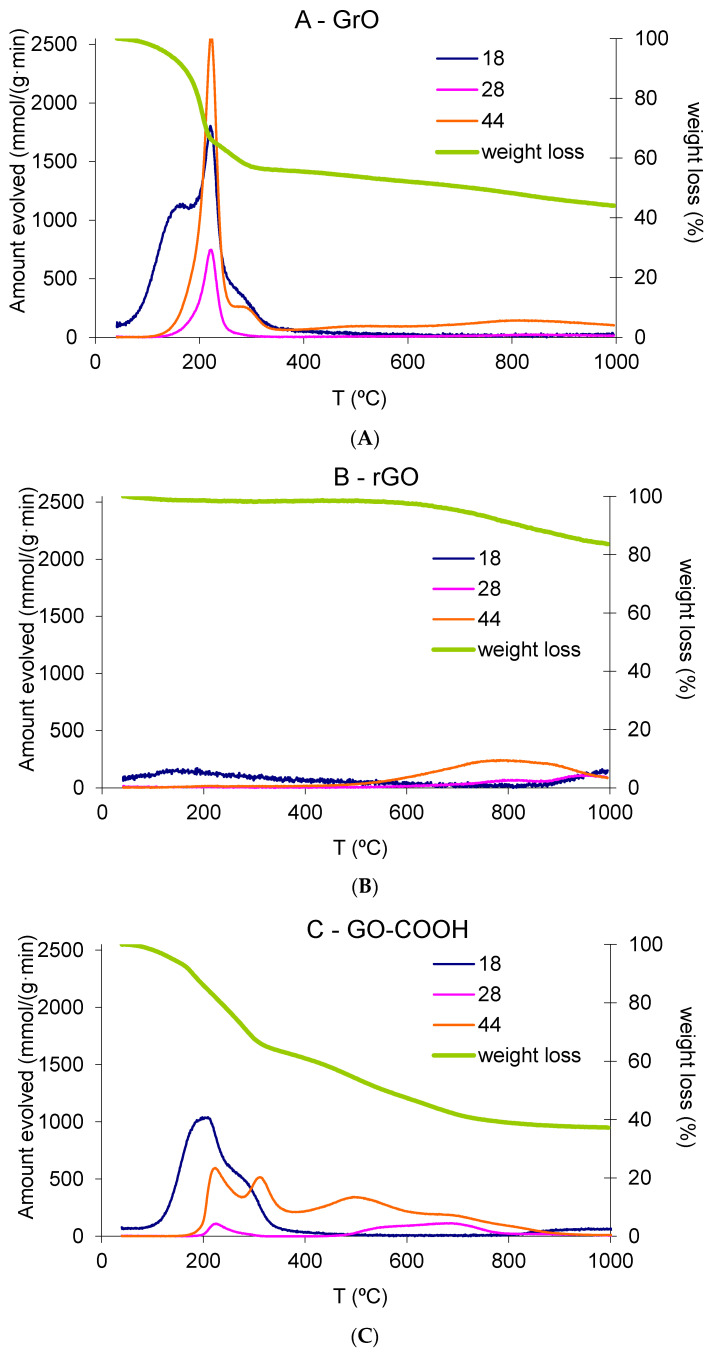
Thermogravimetric weight loss and TGA-MS results for molecular ions of water (18), carbon monoxide (28) and carbon dioxide (44) for (**A**) GrO, (**B**) rGO and (**C**) GO-COOH.

The TG-MS results of powdered GrO, rGO and GO-COOH samples shown in Figure 5A–C, respectively revealing the difference in terms of how KMnO_4_ reacts with a single-layer rGO relative to a graphitic stacked material. Table 3 shows the integrated areas in terms of gravimetric weight loss for MS signals for water, CO and CO_2_.

Figure 5A shows a TGA plot in agreement with those previously reported in the literature for graphite oxide, with an initial sharp decline at around 180 °C and evolution of mainly water and CO_2_, as well as also appreciable amounts of CO (3.6 wt% of initial GrO). A second smoother and minor decrease follows the first drop, centered at 300 °C, with some water and CO_2_ evolved (not CO), as well as the decomposition of organosulfates (see Figure S4) [41]. Most of the weight loss takes place before 400 °C. Figure 5b shows an expected TGA for a reduced graphene oxide sample, with no weight loss until temperatures above 700 °C; beyond this temperature, the weight loss can be attributed to the gasification of the fluffy rGO powder by traces of humidity of the carrier gas. Figure 5C shows very interesting results in terms of both weight loss and gas evolution composition. The equivalent Hummers–Offemann reaction with a single-layer rGO yields a different decomposition pattern than the same reaction carried out with stacked graphite. First, the initial decline presents a similar but slower weight loss (steep), whereas it evolves a similar amount of water and lower amounts of CO_2_ and, especially, CO (lower than 1 wt%). This difference in the slope may be attributed to the fact that GrO is a stacked sample, and the decomposition occurs instantaneously by the confinement of generated vapors between the layers, with explosive behavior and exfoliation taking place. Furthermore, GO-COOH is synthetized from a previously exfoliated sample, and decomposition vapors may evolve freely in a non-explosive manner. Therefore, after the first decrease, there continuous smooth weight loss occurs from around 300 °C to 800 °C, in combination with continuous CO_2_ evolution, with one peak centered at around 310 °C (which may be associated with the second drop in GrO) and another two centered at 500 and 700 °C, respectively. A total of 37 wt% weight loss was quantified as CO_2_ in the whole range of temperatures (20 wt% in the range 400–900 °C), whereas GrO only evolved 22 wt%. Interestingly, water and CO evolution are similar for both GrO and GO-COOH, which means that the population of carboxyl-based groups, such as carboxylic acids, lactones and carboxylic anhydrides (groups varying stability responsible of CO_2_ evolution upon decomposition) are considerably increased. These results are in agreement with our XPS (Figure 3), FT-IR (Figure S2) and ^13^C MAS NMR (Figure 4) characterizations presented above. Table 3 also shows the results of zeta potential for 0.01 mg/mL water suspensions for GO (exfoliated sample result of sonicated GrO) and GO-COOH, with a clear improvement in the overall charge and stability of GO-COOH (zeta potential from −33.2 mV to −48.1 mV for GO and GO-COOH aqueous suspensions, respectively), which is ideal for conjugation with organic substances in biomedical applications. Similar zeta potential resulted were previously reported for carboxyl graphene oxide obtained through chloroacetic acid/NaOH treatment [22].

## 4. Discussion

Our method yielded excellent carboxyl-rich graphene oxide sheets, with the most selective carboxylic acid groups population ever reported (55% carbon-oxygen groups) and without evidence of sheet unzipping, as carbon dots (oxidative debris) were detected over the sheet surface. This material was obtained through a process similar to the Hummers–Offemann process but using a single-layer reduced graphene oxide as a precursor. In this section, we discuss the reaction mechanisms when starting from stacked natural graphite or from a single-layer reduced graphene oxide, with considerable differences revealed in the oxygen group population.

**Figure 6:**
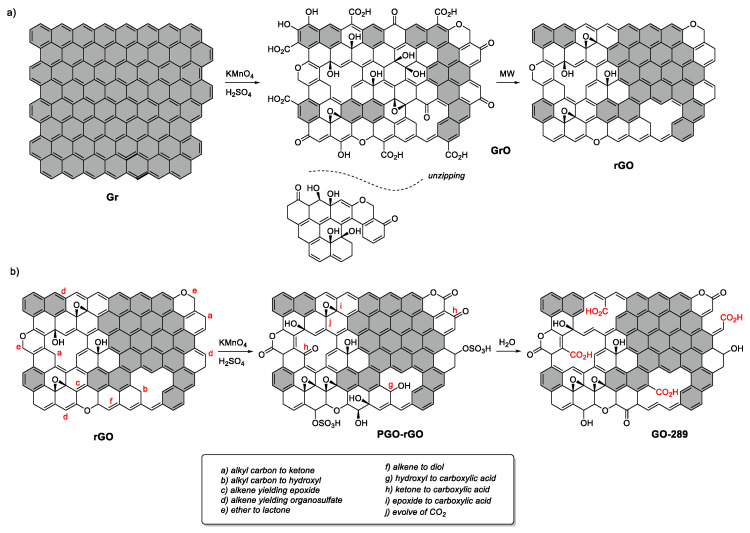
(**a**) Evolution of the graphene layer structure from stacked graphite to graphite oxide (GrO) after the Hummers–Offemann reaction (deep disruption of the delocalized py bonds with the formation of multiple oxygen functionalities, including sheet unzipping) and final graphene oxide (rGO) reduced by thermal treatment (which partially restores some of the py-delocalized cloud and resulting in the appearance of many new defects). (**b**) Evolution of the structure of rGO during the stages of the Hummers–Offemann method. The pristine graphene oxide (PGO) forms new hydroxyls and organosulfates on defects. Water solvation converts these hydroxyls and localized double bonds to carboxyls and anhydrides, respectively, leaving the aromatic domains nearly intact. Letters a to j illustrate the processes taking place during the oxidation and water treatments.

Figure 6A shows the scheme of the transformation of the structure of a single graphene layer; when stacked in graphite, it is converted to graphite oxide and further reduced to graphene oxide under thermal heating. In the first step, the graphene layers are cut and unzipped by the Hummers–Offemann reaction, as evidenced in the unravelling of graphene layers when starting from MWCNT [28,42]. Dimiev et al. [33] demonstrated that the intercalation and previous formation of a sulfuric acid-GIC is necessary for this unravelling. This aspect might explain why small MWCNTs are not unzipped or unraveled, and only shells larger than a certain size are unzipped [32], as is the case with potassium intercalated MWCNT [31]. In the case of small-diameter MWCNT, the graphene layers are overly bent, and the stacking differs considerably compared with that obtained when using graphite, which does not favor the formation of GIC. However, the graphite oxide formation steps occur after the H_2_SO_4_-GIC step [12], the second step of formation of pristine graphite oxide (before unzipping has occurred) and the third step involving solvation in water, which is the final step that eventually produces a vast number of processes, including the final oxidation, oxygen-driven unzipping [13] and defect formation by hydrate-CO_2_ intercalation [43], yielding a net weight increase due to high oxygen uptake (as reported in this work and others [27]). Therefore, if the previous intercalation of sulfuric acid does not take place (step 1), no unzipping occurs, confirming that the unzipping is intercalation-driven [33]. This phenomenon was observed in the present work when carrying out the Hummers–Offemann reaction with a single-layer reduced graphene oxide, without unzipping or high oxidation in the layers (with a net weight loss as opposed to a net weight uptake with respect the parent rGO).

Li et al. [29] demonstrated that unzipping takes place by ring opening of well-arranged epoxy groups. Accordingly, Sun and Fabris [30] indicated (by applying DFT models) that this mechanism of epoxy opening and unzipping was thermodynamically possible, although its rates is considerably limited by oxygen diffusion through the hexagonal carbon pattern of the layer, which must form an adequate arrangement of epoxy groups to induce unzipping. There is possibly no limitation with respect to oxygen diffusion when GIC is formed, followed by PGO (when starting from a stacked graphite structure), yielding graphene layers that are cut and unzipped by a combined intercalation-driven and subsequent oxygen-driven mechanism. However, when no GIC is formed, the starting thermodynamic situation is different, and oxygen diffusion could be considerably lower, making the arrangement of epoxy groups impossible. Consequently, when single-layer reduced graphene oxide (a graphene-based layer with aromatic domains and many defects) is used as a precursor in the Hummers–Offemann method, the following steps take place according to the Dimiev–Tour mechanism [12], as shown in Figure 6B:

No GIC is formed when dissolving rGO in sulfuric acid due to the lack of stacking assemblies, and the graphene precursor is not structurally modified.

The attack of KMnO_4_ (or Mn_2_O_7_/MnO_3_^+^) to rGO takes place in structural defects, in particular in alkyl carbons adjacent to aromatic domains located in both sheet edges, holes or in-plane, yielding ketones, as well as ethers, epoxy, hydroxyl and organosulfates (due to the solvent). Furthermore, localized double bonds are attacked, resulting in their opening and yielding carbonyl groups; some hydroxyl evolve to ketone, and ether evolves to lactone. Aromatic domains remain essentially unchanged.

During water solvation, KMnO_4_ extends its oxidation power due to temperature increase, forming additional carboxyl groups (including carboxylic acids and anhydrides) from ketones and hydroxyls. Some of these carboxyl might evolve as CO_2_ during water solvation, producing holes and defects, which may explain the net solid-phase reduction. Once again, aromatic domains remain the same, and no unzipping takes place.

This is opposite to the high weight increase observed when using graphite as a precursor due to the storage of hydrate CO_2_ in the interlayer, as indicated by Eigler et al. [43]. This hydrate CO_2_ is formed in the water solvation step as a result of the reaction with carbonyls or α-epoxy carbonyls, yielding an intermediate diol, which may be responsible for the C1s XPS contribution centered at 286.7 eV in the GrO sample, as well as the sharp decrease and considerable CO_2_ release during the TG-MS run at 185 °C. On the other hand, the formation of carboxylic acids and even the release of CO_2_ is completed during the water solvation process when using rGO as precursor. This may explain the GO-COOH weight decrease; the difference in ^13^C MAS NMR profile in terms of increased signals for esters and carboxylic acids located at 160 and 170 ppm, respectively; C1s XPS pattern, which is rich in selective carboxyl groups; and, finally, the difference in TG-MS smooth weight loss and CO_2_ evolution along the full range of temperatures due to the decomposition of carboxyl groups with different thermal stabilities, such as carboxylic acids and anhydrides. Additionally, the mobility of oxygen on the graphene skeleton is insufficient; therefore, the obtained graphene sheets are damaged with holes but without the formation of dots due to unzipping.

## 5. Conclusions

The present work yielded remarkable results with respect to improving the carboxylic group content of graphite oxide, representing an interesting strategy to produce a graphene oxide rich in oxygenated groups that may eventually be linked with bioactive molecules. The proposed method could make possible the development of graphene-based drug delivery applications. In addition, several important conclusions can be deduced from the present work:

- Potassium permanganate oxidation in sulfuric acid media presents a very different mechanism when starting from graphenic material with no interlayer assembly. This means that no sulfuric acid-GIC can be formed, and the subsequent reactions follow a mechanism based on oxidation of hydroxyls, alkyl and localized double bonds on carboxylic groups.
- Potassium permanganate oxidation on single-layer graphenic materials does not form carbon dots (or graphene oxide quantum dots). The fact that no intermediate GIC is formed indicated that no cut or unzipping of the graphene sheet occurred.
- Potassium permanganate oxidation in sulfuric acid media with the addition of water yields selectively of more than 55% oxygen functionalities as carboxylic groups with adequately sized sheets, which are useful groups for the potential linking of bioactive molecules, with an improved zeta potential assuring colloidal stability in water.

## Figures and Tables

**Table 1:** Surface elemental relative atomic composition (at-%) of C, O, N and S determined by XPS peak integration, as well as the C/O ratio.

	C	O	N	S	C/O
GrO	64.0	34.0	0.3	1.6	1.88
rGO	90.2	8.8	0.0	0.3	10.25
GO-COOH	70.1	26.3	0.5	2.9	2.67

**Table 2:** Comparison of XPS C1s deconvolution areas (at fixed binding energies) of GO-COOH and other carboxyl graphene oxides reported in the literature. The last column of COOH/total C-O is the ratio of COOH area divided by the sum of the areas corresponding to C-O bonds (sp3C-O+C=O+COOH).

Authors	Treatment	C=C %	sp3-C-C %	sp3-C-O %	C=O %	COOH %	COOH/Total C-O
	Binding energy (eV)	284.6	285.6	286.5	287.6	288.7	%
Guo (2020)	chloroacetic acid/NaOH to GO	63.8		28.7	0.7	6.2	17.4
Kuang (2013)	1-Pyrene carboxaldehyde/NaOH to GO	64.3		17.2	11.3	7.3	20.4
Pan (2014)	Thiourea dioxide/NH3 to GO	41.3	25.4	10.5	12.9	9.9	29.7
This work GO-COOH	KMnO4/H2SO4 to rGO	58.0	9.3	13.4	1.3	18.0	55.0

**Table 3:** Amounts (wt-%) of H_2_O, CO and CO_2_ evolved in the TG-MS experiments, calculated by integration with a standard. The last column shows the zeta potential of 0.01 mg/mL aqueous suspensions with GrO, rGO and GO-COOH.

	TG-MS—Evolved Ions (wt-%)			Zeta Potential
	H_2_O	CO	CO_2_	(mV)
GrO	14.9	3.6	22.1	−33.2
rGO	1	0.3	0.8	-
GO-COOH	14.1	4.9	37.1	−48.1

## Data Availability

Not applicable.

## Supplementary Materials

The following supporting information can be downloaded at: https://www.mdpi.com/article/10.3390/nano12183112/s1, Figure S1: Supplemental TEM.; Figure S2: FTIR spectra of GrO, rGO and GO-COOH; Figure S3: Raman spectra of GrO, rGO and GO-COOH; Figure S4: Additional MS signals of TG-MS runs.
