# Supplementary material for: Effective Method for a Graphene Oxide with Impressive Selectivity in Carboxyl Groups
Source: Nanomaterials (Basel). 2022 Sep 8;12(18):3112. doi: 10.3390/nano12183112 (PMC9500783; doi:10.3390/nano12183112)
Supplement: Supplementary file 1 [file nanomaterials-12-03112-s001.zip › nanomaterials-1908198-supplementary.pdf]

Supplementary Materials

Effective Method for a Graphene Oxide with Impressive Selectivity in Carboxyl Groups

Iluminada Rodríguez-Pastor [1,2], Adelia López-Pérez [1], María D. Romero-Sánchez [1], Juana M. Pérez [3], Ignacio Fernández [3] and Ignacio Martin-Gullon [2,*]

[1] Applynano Solutions, S.L. Alicante Scientific Park #3, 03690 Alicante, Spain
[2] Institute of Chemical Processes Engineering, University of Alicante, 03080 Alicante, Spain
[3] Research Centre CIAIMBITAL, University of Almería, 04120 Almería, Spain
* Correspondence: gullon@ua.es

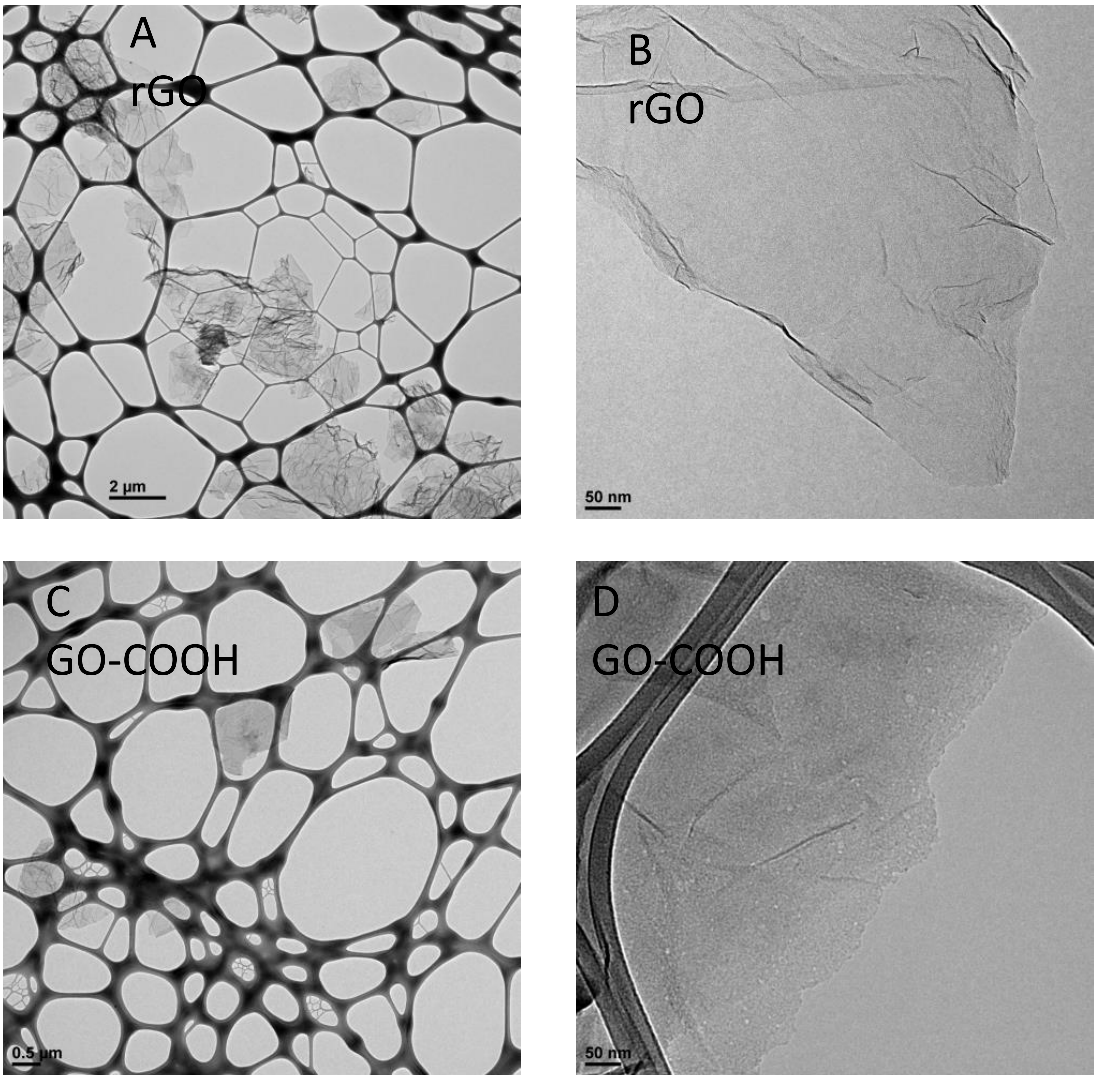

Figure S1. Lower (A, C) and higher magnification (B, D) with respect to TEM of main manuscript, respectively, of rGO and GO-COOH samples. GO-COOH does not present C-dots and present a similar size (comparison A vs. C) and a great number of defects with respect to parent rGO.

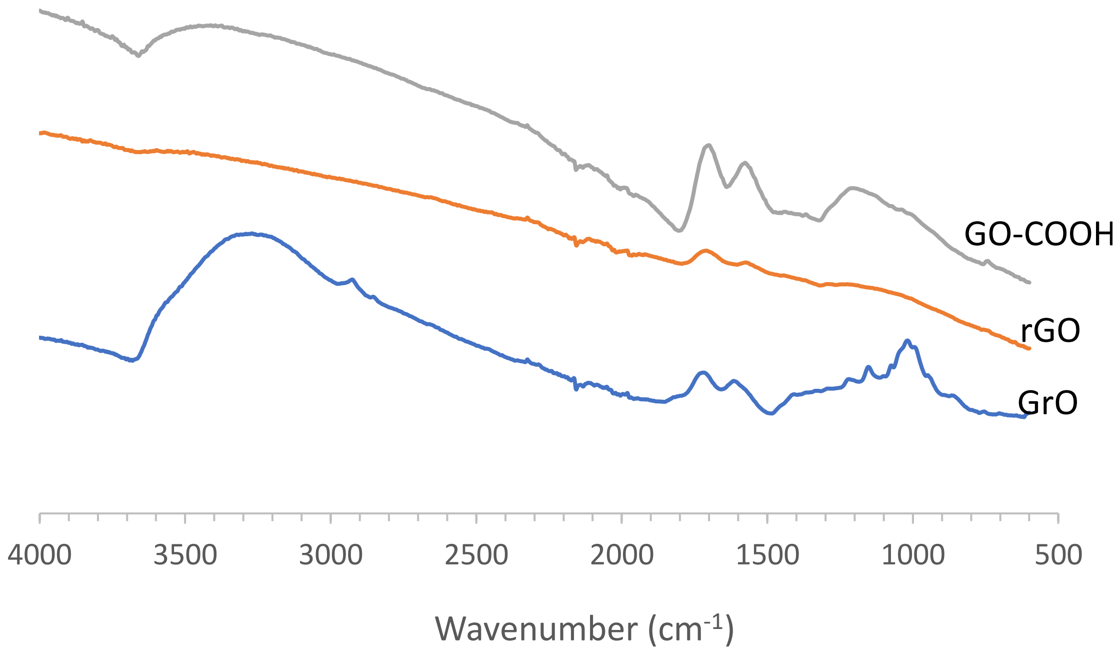

Figure S2. FTIR-ATR of powder samples of graphite oxide GrO, its derived microwave treated reduced Graphene oxide rGO, and carboxyl Graphene oxide GO-COOH derived from rGO. Hydroxyl band (3300 and 1200-1000 cm^{-1}) have more intensity than carbonyl/carboxyl (1700 cm^{-1}) for parent GrO. Upon reduction, most os functionalities are lost, as shown in the flat pattern of rGO, leaving just a tiny reminder of GrO bands. However, potassium permanganate treatment of rGO (GO-COOH) yields higher intensity in carbonyl/carboxyl groups than starting GrO, and lower intensity for hydroxyl groups.

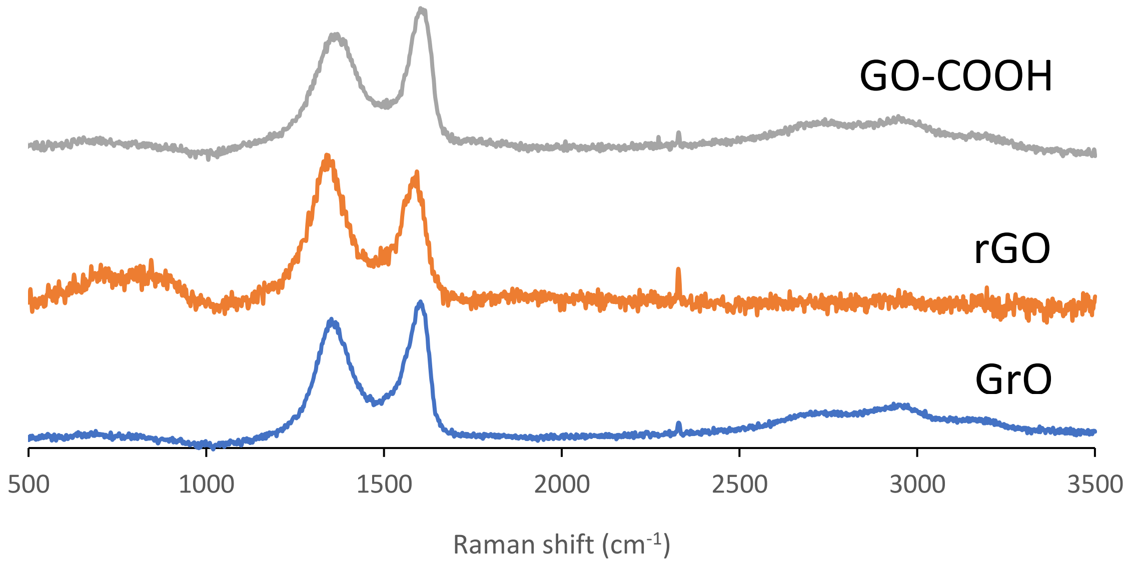

Figure S3. Raman spectra corresponding to powdered samples GrO and its derived rGO and GO-COOH.

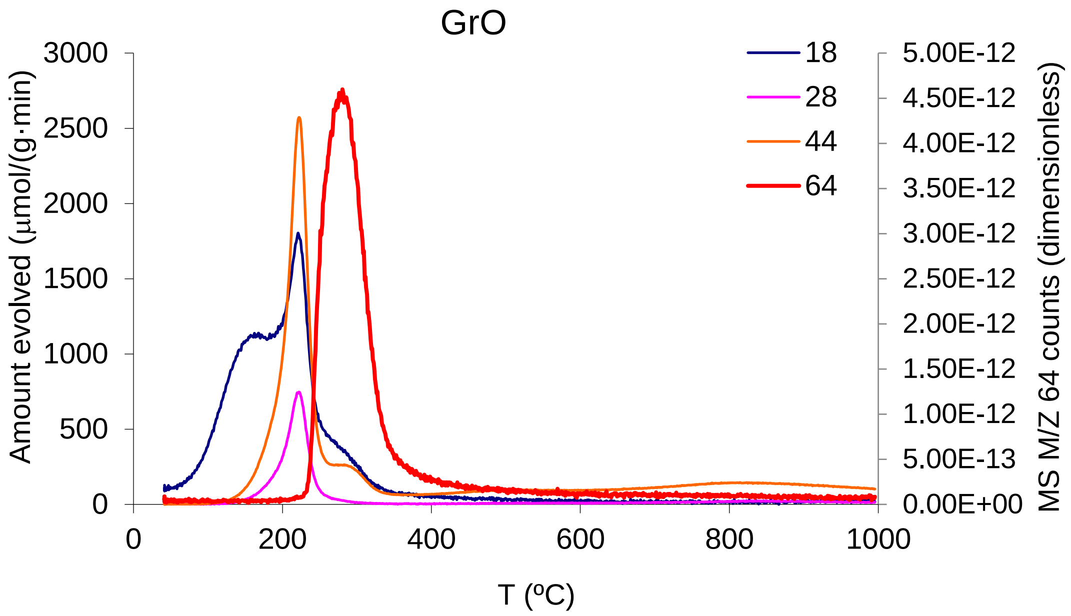

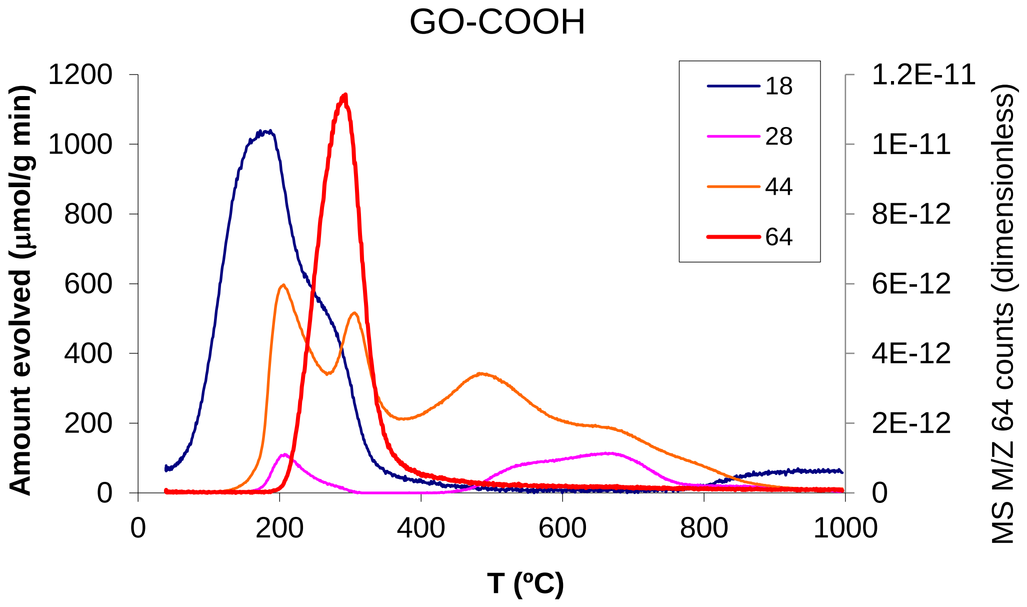

Figure S4. Quantified MS signals of 18 (H_2O), 28 (CO) and 44 (CO_2) plus the non-quantified signal of 64 (SO_2) for GrO and GO-COOH.
